# Complete mitochondrial genome of the *Turbinaria bifrons* (Scleractinia, Dendrophylliidae)

**DOI:** 10.1080/23802359.2021.1948368

**Published:** 2021-07-06

**Authors:** Peng Tian, Jiaguang Xiao, Zhiyu Jia, Xiaolei Wang, Wei Wang, Dingyong Huang, Jianjia Wang, Wentao Niu

**Affiliations:** Laboratory of Marine Biology and Ecology, Third Institute of Oceanography, Ministry of Natural Resources, Xiamen, PR China

**Keywords:** Mitogenome, next-generation sequence, phylogeny, Scleractinia

## Abstract

In this study, the complete mitogenome sequence of the stony coral, *Turbinaria bifrons* Brüggemann, 1877, has been decoded for the first time by next-generation sequencing (NGS) and genome assembly. The assembled mitogenome was 18,880 bp in length, contained 13 protein coding genes, 2 transfer RNAs, and 2 ribosomal RNAs. The complete mitogenome of *T. bifrons* showing 97.09% identities to *Tubastraea tagusensis*. The complete mitogenome provides essential and important DNA molecular data for further phylogenetic and evolutionary analysis for coral phylogeny.

Reef-building coral species of the order Scleractinia play an important role in shallow tropical seas by providing an environmental base for the ecosystem (Fukami et al. 2000). While traditional morphology-based systematics cannot clearly reflect all the evolutionary relationships of Scleractinia, molecular data have, therefore, become increasingly important in recent years to overcome the limitations of morphological analyses among scleractinians (Arrigoni et al. 2017; Terraneo et al. 2017). Data of complete mitochondrial genomes have also become important sources for assessing scleractinian phylogenies due to the declining cost of next-generation sequencing (NGS) technologies (Schuster 2008; Jex et al. 2010; Niu et al. 2018). Nevertheless, there are more than 1600 species, whereas only approximately 100 complete mitogenomes of Scleractinia species have been collected in NCBI (https://www.ncbi.nlm. nih.gov/) to date (Hoeksema and Cairns 2020). *Turbinaria bifrons* Brüggemann, 1877 which belongs to the family Dendrophylliidae is a species usually with gray, green, or brown colors, and with paler calices. This species is mainly distributed from center Indo-Pacific to Japan. In this study, we used NGS technology and *de novo* assembly method to elucidate the complete mitogenome sequence of *T. bifrons* for the first time to provide basic information for further evolutionary and phylogenetic analyses.

The specimen of *T. bifrons* was collected from Yangmeikeng of Daya Bay in Guangdong, China (longitude 114.58569 E, latitude 22.560833N) and it was deposited at Coral Sample Repository, Third Institute of Oceanography, MNR, Xiamen, Fujian, China (contact Tian: tianpeng@tio.org.cn) under the voucher number D40. Total genomic DNA was extracted using the DNeasy tissue Kit (Qiagen, Shanghai, China) and kept at 4 °C for subsequent use. We used NGS to perform low-coverage whole genome sequencing according to Li and Luo (2021). Initially, the raw next generation sequencing reads generated from Illumina Novaseq 6000 platform (Illumina, San Diego, CA). The quality and quantity of data produced by the Illumina sequencing were measured by FastQC (Andrews 2010). After filtering low-quality reads and reads containing adapters and poly-N regions, the obtained clean reads were applied for reconstructing the mitochondrial genome by NOVOPlasty (Dierckxsens et al. 2016) using *Turbinaria peltata* mitochondrial genome (GenBank: NC_024671) as a reference. About 0.08% raw reads (70,834 out of 90,151,010) were *de novo* assembly to produce a single, circular form of complete mitogenome with an average coverage 627×.

The complete mitogenome of *T. bifrons* was 18,880 bp in size and its overall base composition was 25.43% for A, 37.36% for T, 23.67% for G, and 13.53% for C. The protein coding (PCGs), ribosomal RNA (rRNA), and transfer RNA (tRNA) genes of *T. bifrons* mitogenome were predicted by MITOS (Bernt et al. 2013) WebServer (http://mitos.bioinf.uni-leipzig.de/index.py) and then we identified and annotated all genes manually by alignments of homologous mitogenomes with other scleractinians. The complete mitogenome of *T. bifrons* included 13 PCGs, 2 tRNA genes (tRNA^Met^, tRNA^Trp^), and 2 rRNA genes. All PCGs, tRNA, and rRNA genes were encoded on H-strand. PCGs preferred base T, tRNA genes preferred base G, and rRNA genes preferred base A.

The PCGs was 11,835 bp in size, and its base composition was 22.55% for A, 13.85% for C, 23.15% for G, and 40.45% for T. Among all the PCGs, the ND5 had a 11,250 bp intron insertion, and the COI had a 964 bp intron insertion. It was important to note that three PCGs (*ND5*, *ND4L*, and *ND3*) started with GTG codon, *ND6* gene started with ATA codon, the other nine PCGs started with ATG codon. Five PCGs (*ATP6*, *COII*, *COIII*, *ND3*, and *ND5*) were inferred to terminate with TAG, other PCGs terminated with TAA. Among 13 PCGs, the longest one was *ND5* gene (1836 bp), whereas the shortest was *ATP8* gene (216 bp). The number of non-coding nucleotides between different genes varied from 16 to 964 bp. Using BLAST searches in NCBI we found that *T. bifrons* was 97.09% similar to *Tubastraea tagusensis*.

To validate the phylogenetic position of *T. bifrons*, we used MEGA version 7 (Kumar et al. 2016) to construct a maximum likelihood tree (with 500 bootstrap replicates and Kimura 2-parameter model) which contained complete mitogenomes of 24 species derived from Scleractinia. The result shows that *T. bifrons* is closely related to *T. tagusensis*, *Tubastraea coccinea*, *Dendrophyllia arbuscula*, and *Dendrophyllia cribrosa* (Figure 1). In conclusion, the complete mitogenome of the *T. bifrons* deduced in this study provides essential and important DNA molecular data for further phylogenetic and evolutionary analysis for stony coral phylogeny.

**Figure 1.**
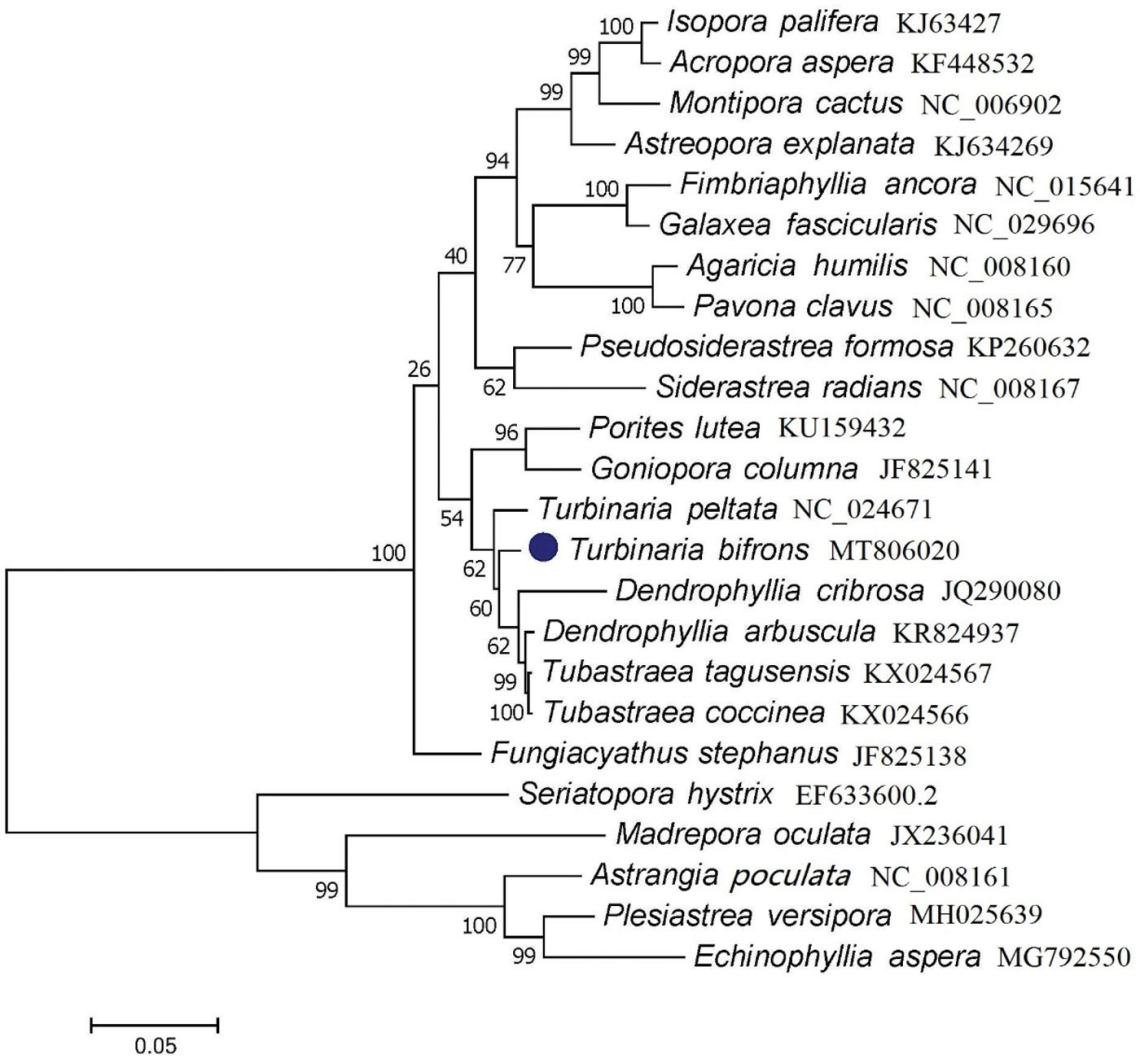
Molecular phylogeny of Turbinaria bifrons and related species in Scleractinia based on PCGs in mitogenome.

## Data Availability

The genome sequence data that support the findings of this study are openly available in GenBank of NCBI (https://www.ncbi.nlm.nih.gov/) under the accession number MH806020. The associated BioProject, SRA, and Bio-Sample numbers are PRJNA737572, SRR14841236, and SAMN19700019, respectively.
